# Fixation Differences in Spatial Visual Perception During Multi-sensory Stimulation

**DOI:** 10.3389/fpsyg.2020.00132

**Published:** 2020-02-05

**Authors:** Jihoon Kim, Ju Yeon Kim

**Affiliations:** ^1^Department of Advertising and Public Relations, University of Alabama, Tuscaloosa, AL, United States; ^2^Department of Interior Architectural Design, Soongsil University, Seoul, South Korea

**Keywords:** visual modality, multi-sensory, eye tracking, fixation, music type

## Abstract

The mood and atmosphere of a service setting are essential factors in the way customers evaluate their shopping experience in a retail store environment. Scholars have shown that background music has a strong effect on consumer behavior. Retailers design novel environments in which appropriate music can elevate the shopping experience. While previous findings highlight the effects of background music on consumer behavior, the extent to which recognition of store atmosphere varies with genre of background music in sales spaces is unknown. We conducted an eye tracking experiment to evaluate the effect of background music on the perceived atmosphere of a service setting. We used a 2 (music genre: jazz song with slow tempo vs. dance song with fast tempo) × 1 (visual stimuli: image of coffee shop) within-subject design to test the effect of music genre on visual perception of a physical environment. Results show that the fixation values during the slow tempo music were at least two times higher than the fixation values during the fast tempo music and that the blink values during the fast tempo music were at least two times higher than the blink values during the slow tempo music. Notably, initial and maximum concentration differed by music type. Our findings also indicate that differences in scan paths and locations between the slow tempo music and the fast tempo music changed over time. However, average fixation values were not significantly different between the two music types.

## Introduction

Music can have diverse effects on consumer behaviors, including emotion (Bitner, 1992; Tansik and Routhieaux, 1999; Eroglu et al., 2005; North et al., 2016), purchasing (North et al., 1999; Alpert et al., 2005), brand attitude and loyalty (Chebat et al., 2001; Grewal et al., 2003), subjective time recognition and time of stay in a store (Holbrook and Anand, 1990), and other behavioral variables (Turley and Milliman, 2000). In the past, consumers automatically accepted the spaces given to them based on the products they chose to purchase. However, people have come to consider emotional experience clues (e.g., store atmosphere) to which they are exposed when selecting products. That is, modern consumers tend to select spaces based on atmosphere rather than merely accepting the spaces that stores create.

In some cases, atmosphere can have a greater effect on a purchase decision than the product itself. Demoulin (2011) found several spatial environmental elements closely related to atmosphere: view, lighting, noise, music, scent, and temperature. Background music is another element of atmospheric impact (Milliman, 1986; Duncan Herrington, 1996). Lusensky (2010) found that background music was the most important element in the experience of a space, and Kim (2016) found that consumers were often exposed to background music, second in frequency only to visual stimuli. Furthermore, visual memory tends to last 1–2 s, whereas auditory memory tends to last 4–5 s, suggesting that humans remember auditory stimuli better than visual stimuli (Finney et al., 2001).

Experimental studies about emotional response to store spaces emerged several decades ago. Using eye tracking to study visual attention, Yarbus (1967) found that scan paths and frequency of attention to a given image varied with task type. More recently, DeAngelus and Pelz (2009) used eye tracking to measure fixation number, fixation duration, and fixation saccade. Greene et al. (2012) extracted fixation data relative to behavioral intention to determine changes over time. These scholars and others have confirmed that eye movement depends on the relative perceived importance of areas in a space. Posner et al. (1980) examined shifts in visual attention among the structures of a visual system. Through visual attention experiments that manipulated attention using signal positioning, they developed a model for attention that incorporated vision and judgment.

Scholars have shown that background music has a strong effect on consumer behavior (Bitner, 1992; North et al., 1999; Chebat et al., 2001; Alpert et al., 2005). While previous findings highlight the effects of background music on consumer behavior, the extent to which recognition of store atmosphere varies with background music type in sales spaces is unknown. To explore this phenomenon further, we analyzed quantitative fixation data for visual stimuli relative to auditory stimuli (i.e., different music genres). In the current study, we investigated (1) eye movement across space by music type, (2) space scan paths by music type, and (3) time between initial concentration and maximum concentration on the space by music type.

## Literature Review

### Cross-modal Sensory Effects on Visual Attention

An important goal for retail managers and marketers is to provide memorable and unique experiences for customers. Multisensory cues can help stores stand out from competitors and attract customers by delivering a pleasant shopping experience (Verhoef et al., 2009; Hagtvedt and Brasel, 2016). Thus, retailers strive to enhance sensory inputs and use emerging digital technology to attract consumers and improve the shopping experience. Especially impactful when used in various combinations, sensory stimuli are basically cues related to vision, audition, olfaction, haptics, and gustation (Spence et al., 2014). Each contributing to subjective interpretations of the world, the five senses enable humans to perceive objects and phenomena in their environment (Calvert, 2001).

While vision is generally assumed to be the dominant sensory mode in retrieving external information (Reda et al., 2013), recent findings in marketing literature suggest that other sensory modalities can moderate visual processing (Hultén, 2011). In particular, sound can impact the primary visual cortex (Shams and Kim, 2010). Neuroscience scholars have found that several sensory brain areas are convergence zones where inputs from different sensory modalities interact, blend, and influence each other (Stein and Stanford, 2008; Murray et al., 2016). For example, multisensory stimuli (e.g., background music heard while finalizing a transaction at a store) can be perceptually clustered together, affecting visual attention, spatial awareness, and emotional state (Driver and Spence, 2000). In this regard, the auditory and visual systems are particularly interlocked. Moreover, sensory cues can non-consciously influence consumer decision making, changing their attitudes and behaviors (Togawa et al., 2019).

Numerous findings support the notion that auditory input during product interaction can strongly influence the perception and evaluation of products (Lv et al., 2020). Compared to an isolated visual stimulus, multisensory cues (i.e., visual-auditory combinations) provide extra assistance in product evaluation. Although scholars have identified how various qualities of store atmosphere might influence customers (Verhoef et al., 2009), the effect of multisensory input from technological advancements on consumer behavior needs further clarification.

### The Effects of Music on Consumer Behavior

Scholars have recognized the effects of using background music to influence consumer behavior in various settings and situations (North and Hargreaves, 1996; Chebat et al., 2001; Alpert et al., 2005). Findings have confirmed that background music offers emotional responses such as pleasure and arousal (Spence et al., 2014).

Scholars have considered the influence of background music on consumers, including the presence or absence of music (Kellaris and Cox, 1989), liked or disliked music (Spangenberg et al., 1996), and manipulating specific components of musical structure. Furthermore, scholars have examined the effects of background music on a wide range of variables, including shopping time (Holbrook and Anand, 1990; Yalch and Spangenberg, 2000), total amount of purchasing (North et al., 1999; Alpert et al., 2005), and overall shopping experience (Dubé and Morin, 2001). More recently, scholars have examined how background music influences consumer perceptions and evaluation of foods. Some have focused specifically on how background music influences the taste of wine (Areni and Kim, 1993) and how background music influences beer taste (Carvalho et al., 2016).

However, research on the effects of background music type (i.e., tempo) on consumer behavior is relatively sparse. Addressing other aspects of background music, scholars have found that quiet (vs. loud) airline cabin noise increased the intensity of sweet solutions and decreased the intensity of umami-flavored solutions (Wang et al., 2015). With regard to tempo, however, few scholars have examined its effect on customer behavior. The way background music tempo influences physiological reactions through the neurophysiology system remains unclear.

### Justification of the Eye Tracking Experiment

People frequently do not choose between alternative courses of behavior; rather, they depend on cognitive abilities that are fairly well developed (e.g., awareness and recognition). Eye movement is one of the quickest and most frequent human actions, allowing people to gather numerous bits of information *via* eye fixations on a perceptual field. Outside that perceptual field, however, detailed information is likely to be missed. Moreover, people are normally unaware of the specific eye movements they make when performing a task. Eye movement is closely associated with covert attention. Covert attention plays a key role in information processing and decision making, not only by acting as a selection device but also by executing and maintaining central processing activity to ensure that decisions occur fast and accurately. Consequently, eye movements not only reflect selective attention but also the intensity and nature of the central cognitive processes in information perception, evaluation, and memory, providing real-time information about these ongoing processes that cannot be obtained otherwise. Thus, measuring where the eye is focused or how the eye is moving can help us understand how individuals view objects.

Data collection using eye tracking experiments does not depend on participant reports or memory. When questioned about their actions, participants might not remember their behavior. They might be unaware of what they did due to forgetting or lack of awareness. By examining data, visualizations, and replays, we can determine causes of behavior without relying on fallible human memory. Eye tracking analysis can lead to discoveries that would be considerably more difficult to uncover using other methods. Eye tracking shows how quickly and for how long customers notice features, key content, and brands and can reveal, through the accumulation of minute changes, where participants spent the most time looking.

Scholars have shown that the human visual system is essential to information searching and decision making (Lee and Ahn, 2012). Because the human visual system automatically completes various functions that are important to goal-directed behavior, visual information has a vital impact on shopping experiences (e.g., product evaluation and purchase). However, few scholars have investigated the role of visual processing using eye tracking devices.

## Materials and Methods

### Design

To test the hypotheses, we conducted a two-condition, randomized, within-subject experiment. The independent variable was music genre (i.e., jazz song with slow tempo vs. dance song with fast tempo), and we measured visual attention using eye tracking technology.

### Stimuli

We conducted a field survey to select the music genres for auditory stimuli. We visited eighty cafés in Seoul, South Korea, and classified the kinds of music they played using 10 genres (i.e., new age, dance, rock, ballade, old pop, electronic, world music, jazz, classic, and hip hop; reference). We chose jazz (Music 1) and dance (Music 2) for our auditory stimuli because these two genres had the highest frequency scores (*N* = 109: jazz = 37; dance = 29; ballade = 23; old pop = 14; no music = 3; electronic = 2; new age = 1; and rock, world music, classic, and hip hop = 0). We used each genre with beat per minute (BPM) to create background music for the experiment (Music 1: jazz song with 88 BMP vs. Music 2: dance song with 137 BMP).

Across different auditory stimulus conditions (i.e., tempo and genre), we collected quantitative data about eye movement and fixation using eye tracking equipment. First, for visual stimuli, we selected a cafe space that participants would recognize as a setting where they might make a purchase. We used a camera (i.e., EOS 600D Crop Body; film size: 22 mm; full frame body: 35 mm; lens: EF-S 10–18 mm, F 4.5–5.6; focal length: 10 mm) to photograph this space and create images for the experiment. We configured the indoor space (approximately 8,000 × 6,000 mm) so that the person placing an order at the counter and the person seated at a table to the side were at adult eye level. As a result, the image was a close approximation of a human field of view (FOV). We also used Adobe Photoshop CS6 to enhance image quality.

### Participants and Procedure

We recruited 40 undergraduate students [20 males and 20 females; mean age of 23.2 years (SD = ± 2.32)] enrolled at a private university in Seoul, Korea, in exchange for extra credit. We invited participants to the lab after confirming that they did not have any vision impairment that might limit their ability to see a computer screen (i.e., naked or corrected vision above 0.5). We prohibited mascara and color lenses during the experiment to maximize the quality of our eye tracking data. Upon arrival to the lab, participants signed informed consent forms approved by the Institutional Review Board (IRB) and then sat down at computer stations.

The distance between the monitor and each participant was roughly 650–700 mm to ensure accurate and consistent eye tracking calibration. Before the experiment, we ran a quick calibration program to prepare the eye tracking camera. On their computer screens, participants watched a white dot move to four different areas. We performed the calibration test up to two times and excluded participants who did not pass. Participants who did pass then read the following instructions on the screen: “Imagine you are visiting a coffee shop. Please look around the café and think how the atmosphere of the space and the music feels.” Because we used a within-subject study design, each participant looked at the image while listening to Music 1 and Music 2; however, the order in which they heard the two auditory stimuli was random. The first multi-sensory stimulation lasted 1 min. After a 1-min rest time, the second multi-sensory stimulation began. The experimental session lasted a total of 3 min and took place in a dark room so that the participants could focus on their task with maximum concentration.

### Dependent Measure

We measured the real-time eye movement of the participants. We used eye tracking equipment (i.e., iMotions: SMI-REDn, 30 Hz) to determine the impact of auditory stimuli on visual perception of the physical environment pictured in the image. We extracted raw fixation data using BeGaze 3.7 (SMI). We set the rate of recording and storage to 30 Hz (i.e., 30 data per second).

## Results

### Effective Rate of Raw Data

From the total sample (*N* = 40), we excluded six participants (3, 11, 14, 23, 30, and 38) who failed to exceed the criterion of 80% eye tracker accuracy and one participant (1) due to technical problems with raw data. Therefore, we analyzed the data from 33 participants. The mean effective rate was 90.9% (SD = ±4.13) (see Table 1). In the experiment, we set the fixation time for the visual stimuli to 120 s (i.e., 60 s with Music 1 and 60 s with Music 2). Therefore, we analyzed 3,600 (120 s × 30 Hz) fixation data for each participant.

**Table 1:** Participant tracking ratio.

Value	*N*	Mean	SD	*t*	*p*
All participant	40	−1.61	15.11	−0.67	0.715
80% or less excluded participants	33	−0.76	4.13	−1.07	0.000^**^

** *p < 0.05*.

### Raw Data Extraction

Using the eye tracking data, we extracted values for fixations, saccades, and blinks, the primary components of human eye movement. Fixations occur when the eyes stop scanning and hold in one place for a time while saccades are the rapid eye movements that occur between fixations, allowing the fovea to shift from one point of interest to another. When plotted in chronological order, fixations and saccades constitute a scan path. Fixation count refers to the number of fixations that occurred in an area of interest every 0.03 ms, indicating how long an individual spent looking at a particular part of the image on the screen (i.e., degree of interest). Frequency refers to how many times per second the eye tracker registered the position of the eyes (i.e., raw data divided by fixation count). Blinking refers to the involuntary act of shutting and opening the eyelids. During each blink, the eyelid blocks the pupil and cornea from the illuminator, resulting in missing raw data points at particular *x*-*y* coordinates.

Fixation and saccade frequencies did not differ significantly between Music 1 and Music 2. Fixation frequency for Music 1 (12.29) and Music 2 (12.22) was close. However, blink frequency for Music 2 (12.29) was twice the frequency for Music 1 (6.28). The fixation and saccade frequency and mean values were not significantly different between Music 1 and Music 2, but the blink frequency and mean values for Music 2 were two times those of Music 1 (see Table 2). In addition, although the fixation mean values were similar, the fixation standard deviation for Music 1 was higher than Music 2.

**Table 2:** Average count and frequency of raw eye tracking data for all participants.

	Music type	*M*	SD	Count	Frequency
Fixation	1	1451.94	125.71	122.03	12.29
	2	1458.03	79.35	123.3	12.22
Saccade	1	146	30.73	106.7	1.37
	2	148.94	26.41	108	1.38
Blink	1	168.42	95.99	26.76	6.28
	2	1451.94	88.5	25.36	12.29

### Analysis of Eye Gaze Pattern Differences

Paired sample *t*-tests for fixation (*t* = 0.26, *p* = 0.82) and frequency (*t* = 0.10, *p* = 0.92) indicated no significant difference between Music 1 and Music 2. However, analysis of variance (*s*^2^) for fixation revealed that the standard deviation for Music 1 (*s*^2^ = 15,804) was more than two times Music 2 (*s*^2^ = 6294.78). Therefore, although the mean values were similar, fixation values differed across participants by music type (see Table 3). Moreover, although fixation values differed across the participants, changes in fixation over time and the order of fixations did not. Therefore, we analyzed individual fixation data in more detail.

**Table 3:** Mean, population variance (*σ*^2^), and standard variance (*s*^2^) for raw eye tracking data by music type.

	Music type	*σ*^2^	*s*^2^
Fixation	1	15325.09	15,804
	2	6105.97	6296.78
Count	1	390.51	402.72
	2	388.94	401.09

**Figure 1:**
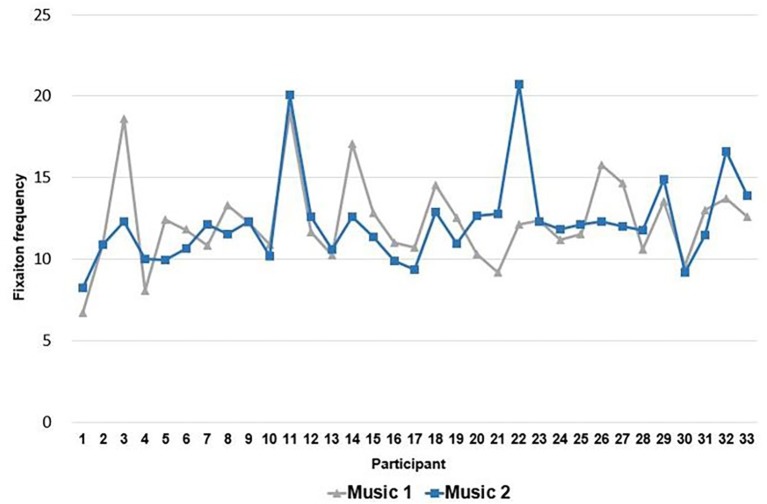
Fixation frequency for each participant.

We compared raw fixation data, count, and frequency for Music 1 and Music 2. According to the raw data, the number of participants (*n* = 18) who showed more fixation during Music 1 was higher than the number of participants (*n* = 15) who showed more fixation during Music 2. In terms of fixation count, the number of participants (*n* = 17) with a higher index during Music 2 was higher than the number of participants (*n* = 14) with a higher index during Music 1. Two participants had the same fixation count during Music 1 and Music 2. The number of participants (*n* = 14) with higher average frequency for Music 1 was the same as the number of participants (*n* = 14) with higher average frequency for Music 2 (see Figure 1). Five participants had the same average frequency during Music 1 and Music 2.

### Differences in Initial Concentration and Maximum Concentration

We defined the time at which the top 5% index value for high fixation started as initial concentration and the time at which the index value for high fixation peaked as maximum concentration. Results of paired *t*-tests for average initial concentration (*t* = 1.46, *p* = 0.15) and average maximum concentration (*t* = 0.23, *p* = 0.82) revealed no significant difference between Music 1 and Music 2.

On average, initial concentration began at 12 s during Music 1 and 15 s during Music 2. Based on population variance (*σ*^2^) and sample variance (*s*^2^) values among the participants, initial concentration during Music 1 was 1.5 times faster than during Music 2. Maximum concentration began at 36 s during Music 1 and 37 s during Music 2, and differences among the participants were not significant between Music 1 and Music 2. Although differences in average initial concentration were not large, differences in variance indicate different initial concentrations among the participants.

**Figure 2:**
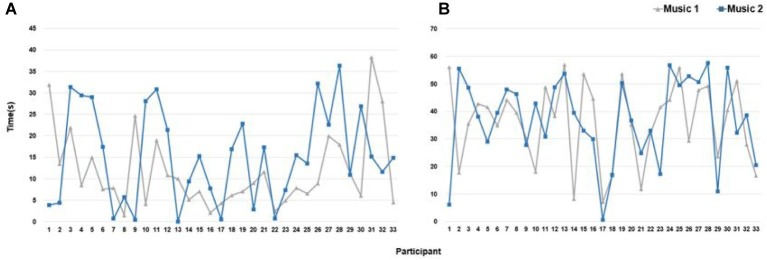
Concentrated eye movement track for each participant during Music 1 and Music 2. **(A)** Initial concentration times. **(B)** Maximum concentration times.

The number of participants (*n* = 22) with earlier initial concentration during Music 2 was twice the number of participants (*n* = 11) during Music 1. The average initial concentration was between 10 and 15 s depending on music type, and the difference in average initial concentration between Music 1 and Music 2 was at least 5 s. Maximum concentration also differed between Music 1 and Music 2. The number of participants (*n* = 19) with an earlier maximum concentration during Music 1 was higher than the number of participants (*n* = 14) during Music 2. Maximum concentration differed among the participants (see Figure 2).

**Figure 3:**
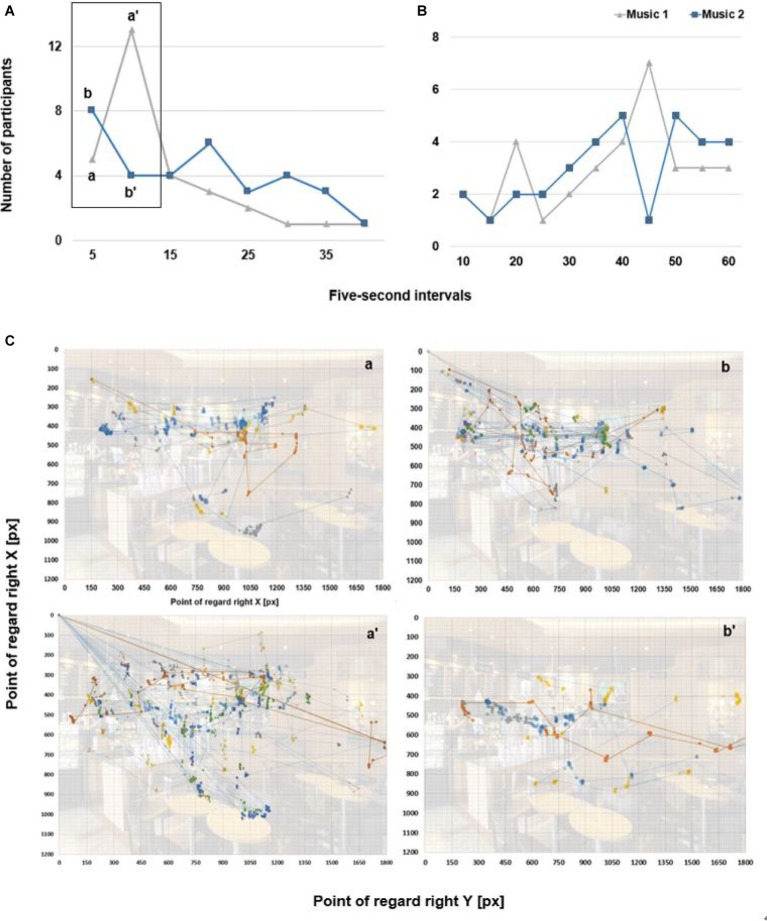
The time series for the number of participants on concentrated fixation. **(A)** Initial concentration at 5-s intervals. **(B)** Maximum concentration at 5-s intervals. **(C)** Spatial fixation points of extracted number of participants. (**a**) Participants (*n* = 5) with Music 1. (**a′**) Participants (*n* = 13) with Music 1. (**b**) Participants (*n* = 8) with Music 2. (**b′**) Participants (*n* = 4) with Music 2.

We analyzed initial concentration frequency at five-second intervals. In the first interval, the number of participants (*n* = 8) with initial concentration during Music 2 was higher than the number of participants (*n* = 7) during Music 1. In the second interval, the number of participants during Music 1 (*n* = 13) was more than three times the number of participants (*n* = 4) during Music 2. After 20 s, the number of participants who began concentrating during Music 2 was higher. We then analyzed maximum concentration frequency at 5-s intervals. We found no difference in the number of participants in the first two intervals. However, at 20 s, the number of participants (*n* = 4) during Music 1 was higher than the number of participants (*n* = 2) during Music 2. The difference between Music 1 (*n* = 7) and Music 2 (*n* = 1) was larger at 45 s (see Figure 3). At all other times, the number of participants during Music 2 was higher than or equal to the number of participants during Music 1.

### Differences in Scan Paths and Locations

**Figure 4:**
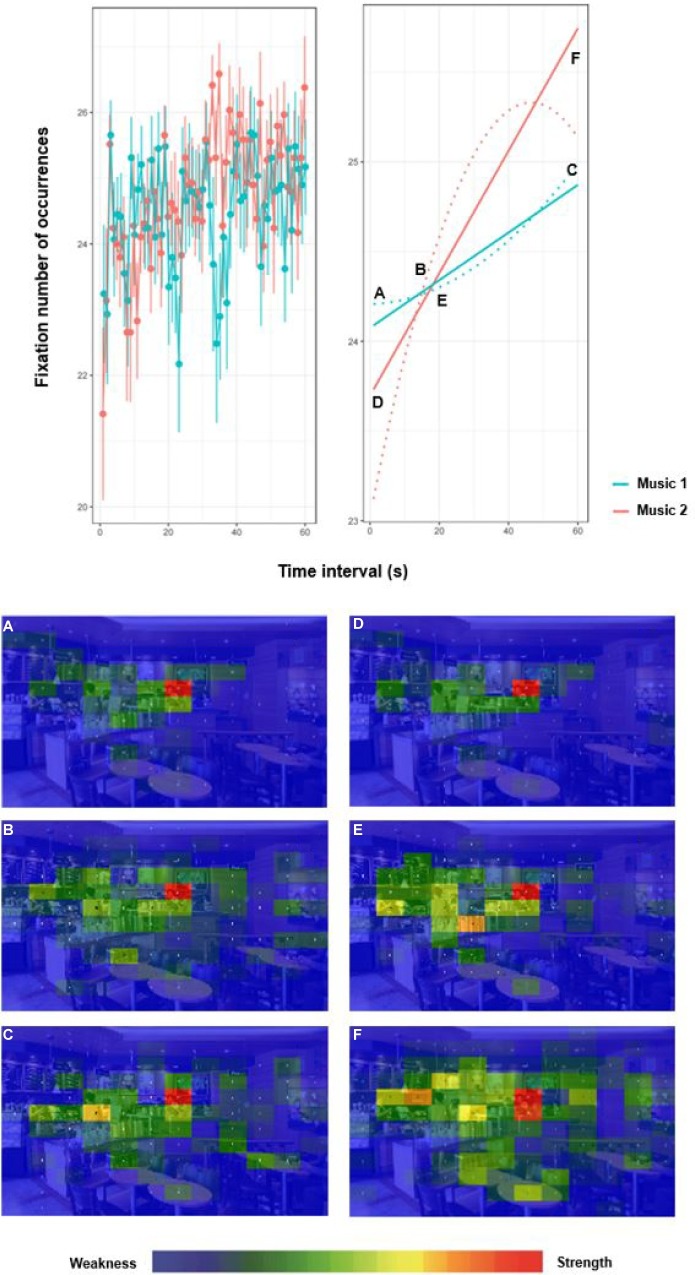
Fixation number of occurrence at time intervals during Music 1 and Music 2. Gridded AOI fixation **(A–F)** with color spectra.

To determine areas of the screen in which participants showed interest, we divided the visual stimulus into a 12 × 12 grid (see Figure 4). We used color spectra to indicate relative degrees of visual interest across the Gridded Area of Interest (AOI).

We divided areas of interest into three time ranges – early period (T1: 0 ~ 20 s), middle period (T2: 20 ~ 40 s), and late period (T3: 40 ~ 60 s) – and compared degrees of interest between Music 1 and Music 2. Analysis of grids during Music 1 revealed that exploration of the entire image was not intensive in T1 and that the primary area of interest was e-g’ (i.e., the employee making coffee). The distribution of eye movement was primarily in the left half of the image. In T2, areas of exploration were more spread out to the left and right of the primary area of interest in T1. In T3, the area of interest shifted from e-g’ to g-e’ (i.e., product display). Between 10 (i.e., early period; highest frequency of initial concentration) and 45 s (i.e., late period; highest frequency of maximum concentration), the area of interest shifted from human objects to purchasable objects.

Analysis of grids during Music 2 revealed that initial concentration began in T1 and that frequency decreased thereafter (see Figure 4). As we found during Music 1, the primary area of interest was e-g’ in T1 and began to shift in T2. In T3, maximum concentration occurred on e-g’ and d-c’ (i.e., upper wall of the cafe where the coffee menu is visible).

## Discussion

Traditionally, store managers have focused on interior store aesthetics for visual stimuli. To a lesser extent, they have started to pay attention to auditory stimuli. Previous findings have shown how cross-modal sensory stimuli can affect consumer behavior (Togawa et al., 2019). Recently, store marketers have increasingly used multisensory cues in their stores to create positive and emotional experiences for customers. Exploring the effect of multisensory stimuli on consumer behavior offers a more holistic perspective on the atmospheric features to which customers are likely to be exposed in a retail environment.

In the current study, we analyzed differences in visual exploration and fixation by music type. First, the average fixation values did not differ significantly between Music 1 and Music 2. One possible explanation is that because visual cues trigger our dominant sense, auditory cues might not exert a strong influence on eye movement. However, according to analysis of variance, the fixation values for participants during Music 1 were at least two times higher than during Music 2, and the blink values during Music 2 were at least two times higher than during Music 1. Comparing frequencies of eye movement between the raw data and the count values, we found that the areas across which eyes moved were larger during Music 2 (i.e., fast tempo), but that fixation frequency was higher during Music 1 (i.e., slow tempo). This analysis is meaningful because objects in scenes are generally coded for visual attention because they provide external information (Navalpakkam and Itti, 2005). Through their visual attention, people prioritize these perceptual objects based on their relative perceived importance. These visual attention patterns are reflected in eye movement. Thus, we inferred covert cognitive processes from eye tracking data. Moreover, in our analysis of data, rather than average differences by music type, we examined relative mean values between fixation and count. In addition, because fixation patterns varied across music type and participants, we had to consider each factor separately.

Second, initial and maximum concentration differed by music type. Regardless of music type, maximum concentration began after at least 40 s on average. Initial concentration was earlier during Music 1, and variance among participants was larger during Music 2. These findings suggest differences in the way the participants responded to music. The average maximum concentration was 36 s during Music 1 and 37 s during Music 2, and the earliest maximum concentration was 35 s. When we divided the time into 5-s intervals and examined frequency of initial concentration across the participants, we found that initial concentration began in the early period for both types of music. However, during Music 1, frequency of initial concentration was high at 10 s (39%) in T1. During Music 1, the highest frequency of maximum concentration was 45 s (21%), but a high frequency also emerged at 20 s (12%) in T1.

Third, our findings indicate that differences in scan paths and locations between Music 1 and Music 2 changed over time. When we divided the viewing time into T1, T2, and T3, we found that eyes moved to the menu or products on the wall in T1. In T2, scan paths differed between music types. During Music 1, scan paths dispersed across the entire image so that eyes landed on both humans and products. During Music 2, scan paths dispersed across the entire image in T3, later than during Music 1. Synchronized stimuli, resulting in cross-modal effects (i.e., visual and auditory), present different routes for perceiving objects in the scene. Auditory cues, enabling cross-modal sensation, further explain how individuals view objects. The results show how auditory cues might interact with visual cues. As sensory marketing research shifts attention from single sensory systems to cross-modal sensory systems, scholars need better insight into the way various sensory systems might interact with each other.

In the current study, we analyzed differences in eye movement and fixation during multi-sensory stimulation (i.e., screen image and background music). Although differences in average eye-movement values between music types were not large, the results are meaningful because we were able to extract the characteristics of visual perception and degree of interest in spaces using eye tracking. Our findings should guide practitioners of neuro-marketing to consider multi-sensory stimulation when designing spaces where consumers are likely to purchase products.

### Limitations of the Study

The limitations of the current study open pathways to future research. First, we exclusively recruited college students. Scholars should consider using wider, more random sampling in order to increase generalizability. Second, we focused on two different genres of music: jazz music with slow tempo and dance music with fast tempo. Given the variety of existing genres, scholars should consider examining the potential impact of other types of background music. Third, we conducted this study conducted in a laboratory setting rather than investigating consumers in their normal shopping environments. For visual stimuli, we selected a cafe space that participants would recognize as a setting where they might make a purchase. Then we created images for the experiment. Participants in a real-setting experiment might have responded to the questionnaire differently. Follow-up field research could more fully reveal the effect of background music on the perceived atmosphere of a service setting. Finally, scholars should qualitatively examine how consumers move their eyes across a visual field and where they place their attention in the field of view.

## Conclusion

Sensory information (e.g., sights, sounds, smells) is typically stored in and retrievable from a sensory memory location. Sensory memory intensifies with the repetition of sensory information, serving as a buffer for external sensory stimuli. The perception of sensory attributes occurs when the process satisfies the condition. Attention then increases with the activation of a particular sensory modality (e.g., visual or auditory). Nearly 70% of the sensory receptors in humans are allocated to vision, making it the most important element in the human sensory system. Thus, understanding how humans process visual sensory information and how it interacts with other sensory information is important. To investigate the effect of background music on visual perception of a physical environment, we conducted an eye tracking experiment in a service setting.

Although we did not find any significant differences between the slow tempo and fast tempo conditions with regard to visual perception and interaction, the individual differences between groups by music type are meaningful. Results show that the blink values during the fast tempo music were at least two times higher than the blink values during the slow tempo music. Moreover, eye movement was more active when the background music tempo was relatively fast, indicating that external auditory information might have activated the visual cortex. We also investigated how differences in scan paths and locations between the slow tempo music and the fast tempo music changed over time. Our findings provide an empirical basis for examining changes in the way consumers respond to multi-sensory stimuli.

Applying Yarbus (1967) to the current study, we shed further light on differences in visual perception during multi-sensory stimulation in a service setting. We measured visual attention using eye tracking technology and compared various components, including fixation, saccade, and blink. Our findings suggest that a qualitative research method (e.g., interviews) could capture the interaction or joint effect of multi-sensory cues on visual perception.

## Data Availability Statement

The datasets generated for this study are available on request to the corresponding author.

## Ethics Statement

The studies involving human participants were reviewed and approved by IRB Office, Soongsil University. The patients/participants provided their written informed consent to participate in this study.

## Author Contributions

JK and JYK contributed to the conception and design of the study. JK organized the database and analyzed and interpreted the data under the supervision of JYK. All authors contributed to the writing and editing of the manuscript.

### Conflict of Interest

The authors declare that the research was conducted in the absence of any commercial or financial relationships that could be construed as a potential conflict of interest.
